# Helicobacter pylori Infection: Prevalence, Risk Factors, and Treatment Efficacy in Symptomatic Patients in Zakho City, Kurdistan Region, Iraq

**DOI:** 10.7759/cureus.73873

**Published:** 2024-11-17

**Authors:** Ali Y Saeed, Brisik H Rashad, Bakhtyar N Ali, Ahmed H Sulaivany, Khalid S Ibrahim

**Affiliations:** 1 Department of Biology, College of Science, University of Duhok, Duhok, IRQ; 2 Department of Biomedical Sciences, College of Medicine, University of Zakho, Zakho, IRQ; 3 Department of Gastroenterology, Health Directorate of Zakho, Duhok, IRQ; 4 Department of Biology, College of Science, University of Zakho, Zakho, IRQ

**Keywords:** h. pylori, iraq, kurdistan region, risk factors, urea breath test

## Abstract

*Helicobacter pylori* (*H. pylori*) is a globally prevalent bacterium, infecting roughly half the global population, with higher prevalence rates in developing countries. This study aimed to investigate the prevalence of *H. pylori* among symptomatic dyspeptic patients in Zakho City, Iraq, evaluate its association with various risk factors, as well as evaluate the effectiveness of treatment in curing this bacterium. Of a total of 150 dyspeptic patients, 50 who had received antibiotics were excluded, leaving 100 patients without antibiotics enrolled in this study. These participants, aged 11-67 years, visited the private Nawroz Laboratory in Zakho City, Kurdistan, Iraq, between June 2021 and October 2022. These patients were tested using the Helicoprobe 14C-Urea breath test and data on various factors, including age, gender, smoking, family size, drinking water source, education level, BMI, hemoglobin levels, and blood group, were collected through structured interviews. In this study, the prevalence of this bacterium was 50%, with no significant difference observed between males and females as well as BMI, smoking, source of drinking water, and blood groups while significant associations were found between infection and increasing age, low Hb levels, and educational level. Notably, 46.7% of patients failed to respond to standard triple therapy, possibly due to antibiotic resistance. The ineffectiveness of standard triple therapy for *H. pylori *highlights the need for tailored treatments based on local antibiotic resistance patterns to improve prevention and treatment strategies with further investigation studies.

## Introduction

It is estimated that half of the world’s population, from both developed and developing countries, is infected by *Helicobacter pylori (H. pylori)*. However, its incidence is higher in developing countries than in developed countries [1]. Several global studies have highlighted that the prevalence of *H. pylori *infection may be associated with several risk factors such as age, socio-economic status, poor hygiene, overcrowding of family members, smoking, blood group O, high body mass index (BMI), and iron deficiency [2]. Despite its high prevalence, the majority of people are asymptomatic, and only 10-15% of them can be symptomatic and manifest as peptic ulcers and gastric malignancy [3]. Stefano et al. reported that although the exact mechanisms of transmission are still unclear, oral-to-oral or fecal-to-oral transmission remains the most prevalent way of infection [4]. Regarding the laboratory diagnosis of* H. pylori*, both invasive and non-invasive methods are used. Invasive methods include culture, histopathology, rapid urease test, and PCR techniques requiring biopsy samples [5] while non-invasive methods include stool antigen test, urea breath test, and serology [4]. However, researchers have highlighted that among both invasive and non-invasive methods, only tests with sensitivity and specificity greater than 90% are reliable and recommended [6]. Several studies have emphasized that serological tests are not accurate for the diagnosis of active H. pylori infection and cannot differentiate between past and cured H. pylori infection, as well as being characterized by low specificity [2]. As a result, a high immunoglobulin G (IgG) titer lasts for years after eradication of the infection. It has been confirmed that the urea breath test is considered the gold standard among non-invasive methods and is characterized by high sensitivity and specificity [7].

Treatment of *H. pylori *infection is necessary in order to cure peptic ulcers and to prevent further consequences such as gastric cancer [8]. Guidelines for *H. pylori *therapy are continuously changing due to the emergence of drug-resistant bacteria [9]. It has been previously recorded that the standard triple therapy (proton pump inhibitor, amoxicillin, and clarithromycin or metronidazole) is less effective in our area due to resistance to both clarithromycin and metronidazole [10].

Thus, the first-line triple therapy, consisting of proton pump inhibitors (pantoprazole or esomeprazole), amoxicillin, and levofloxacin for a duration of 14 days has been adopted. However, a recent study by Kim and Chung emphasized that the best treatment is tailored therapy based on the results of antibacterial susceptibility tests performed in the area [11]. Therefore, this study is undertaken to assess the prevalence of *H. pylori* among symptomatic dyspeptic patients and its relationship with certain risk factors, as well as to evaluate the effectiveness of the treatment against this pathogen.

## Materials and methods

Study duration and location

This cross-sectional study was conducted at the Private Nawroz Laboratory in Zakho City, Iraq, from June 2021 to October 2022. Zakho city is located in Duhok Governance, Kurdistan region of Iraq, and its population is around 350,000 people. It is situated at around 37.15 °N and 42.68 °E and has close borders with Syria and Turkey.

Inclusion and exclusion criteria

A total of 150 patients (51 males and 99 females) suffering from dyspepsia and epigastric pain, who sought treatment at Private Nawroz internal medicine clinics, were initially enrolled in this study. However, 50 patients were excluded from the study because they had received antibiotics within one week of their visit. Consequently, 100 patients (31 males and 69 females) ranging in age from 11 to 67 years with a mean age of 29.77 ± 10.82, were selected for the study.

Each participant underwent an interview, and a structured questionnaire was used to collect data on the relevant factors. The factors studied included age, gender, smoking status, hemoglobin level, body mass index, drinking water, family size, educational level, and blood group. Inclusion criteria required patients to have abstained from proton pump inhibitors and antibiotics for at least two weeks and to have fasted for four to six hours before the study.

To estimate body mass index (BMI), we divided each patient's weight in kilograms by their height in meters square. Patients were classified as underweight (≤ 18.5), normal weight (18.5 - 24.9), overweight (25-29.9), and obese or severely obese (>30).

The ABO and Rh blood groups were determined for each patient using the conventional hemagglutination test with monoclonal anti-A, anti-B, and anti-D antibodies (Torax Biosciences, Newtownabbey, United Kingdom). To determine hemoglobin (Hb) levels, one milliliter of blood was collected from each patient in sterile ethylenediaminetetraacetic acid (EDTA) tubes, thoroughly mixed, and processed using the Swelab Hematology Analyzer machine (DS Biomed, Kemah, Texas, US).

Urea breath test

The Helicoprobe 14C-Urea breath test was used in this study. The patient swallowed a 14C-labelled urea-containing capsule (HeliCap, Mayoly Spindler, France) with water. The capsule's radioactivity is 1 µCi (37 KBq), which is considered safe for humans. After 15 minutes, the patients exhaled into a cartridge (Helicoprobe breath card, Kibion, Upsala, Sweden) through its mouthpiece until the indicator color changed from orange to yellow. The breath card was then inserted into a specialized small desktop Geiger-Muller counter (Helicoprobe-analyzer, Noster system AB Stockholm, Sweden) and the radioactivity was counted for 250 seconds. The results were displayed on the LCD screen in a digital form, which included 0: patient not infected, 1: borderline result, and 2: patient infected. These results corresponded to radioactivity as count per minute as follows: <25 CPM: patient not infected, 25-50 CPM: borderline result, and >50 CPM: patient infected. Grades 0 and 1 were considered negative while grade 2 was considered positive.

Ethical approval

Ethical approval was obtained from the Research Ethics Committee of Duhok Directorate General Health No. 13072022-7-7.

Statistical analysis

Data analysis was performed using GraphPad Prism (version 9.4.1; GraphPad Software Inc., La Jolla, California, US). The chi-squared test was applied with a significant level of a p-value of <0.05. Spearman’s correlation method was used to assess the nonparametric correlation between age, BMI, and the frequency of *H. pylori* infection. Additionally, multiple linear regression was conducted, and the model fit for the logistic regression analysis was evaluated using the Hosmer-Lemeshow test for these factors.

## Results

The prevalence rate of *H. pylori* infection was 50% among the studied patients, with 14/31 males (45.2%) and 36/69 females (52.2%) infected. There was no significant association between gender and *H. pylori* infection (p < 0.31) (Table 1).

**Table 1:** Risk factors and their association with Helicobacter pylori infection

Factors	Total number	No. of positive (%)	No. of negative (%)	p value
Age				
20-10	19	7 (36.8)	12 (63.2)	<0.0001
21-30	39	20 (51.3)	19 (48.7)	
31-40	29	12 (41.4)	17 (58.6)	
41-50	8	5 (62.5)	3 (37.5)	
51-60	3	2 (66.7)	1 (33.3)	
>61	2	2 (100.0)	0 (0.0)	
Sex				
Male	31	14 (45.2)	17 (54.8)	0.3197
Female	69	36 (52.2)	33 (47.8)	
Hb				
Normal	73	32 (43.8)	41 (56.2)	0.001
Low	27	18 (66.7)	9 (33.3)	
BMI				
Normal	54	26 (48.1)	28 (51.9)	0.5969
Overweight	25	13 (52.0)	12 (48.0)	
Obese	14	7 (50.0)	7 (50.0)	
Underweight	7	4 (57.1)	3 (42.9)	
Drinking water				
Tap water	62	33 (53.2)	29 (46.8)	0.2007
Filtered water	38	17 (44.7)	21 (55.3)	
Educational level				
Illiterate	32	22 (68.8)	10 (31.3)	<0.0001
Primary school	15	6 (40.0)	9 (60.0)	
High school	44	20 (45.5)	24 (54.5)	
University	9	3 (33.3)	6 (66.7)	
Family member				
2	1	0 (0.0)	1 (100.0)	<0.3122
3	3	3 (100.0)	0 (0.0)	
4	17	8 (47.1)	9 (52.9)	
5	10	4 (40.0)	6 (60.0)	
6	14	8 (57.1)	6 (42.9)	
≥ 7	55	27 (49.1)	28 (50.9)	
Smoking				
Yes	19	8 (42.1)	11 (57.9)	0.3182
No	81	40 (49.4)	41 (50.6)	
Blood group				
O +ve	50	27 (54.0)	23 (46.0)	0.2171
O -ve	6	3 (50.0)	3 (50.0)	
A +ve	25	10 (40.0)	15 (60.0)	
A -ve	2	1 (50.0)	1 (50.0)	
B +ve	13	7 (53.8)	6 (46.2)	
AB +ve	6	2 (33.3)	4 (66.7)	
After treatment	30	14 (46.7)	16 (53.3)	0.4772
Without treatment	70	36 (51.4)	34 (48.6)	

Regarding age groups, the prevalence of infection was highly significant (p < 0.0001). The lowest percentage (36.8%) was recorded in the 10-20-year age group while the highest percentage (100%) was in the age group of > 61 years. A highly significant association (p < 0.0001) was also observed between infection and haemoglobin (Hb) levels; 18 out of 27 patients (66.7%) with low Hb tested positive versus 32 out of 73 (43.8%) with normal Hb levels. There was no statistically significant association (p < 0.59) between infection status and BMI categories (normal, underweight, obese, or overweight) (Table 1). Additionally, no significant associations were found between infection status and water status (tap vs. filtered water) (p <0.20), family density (p <0.31), smoking status (p <0.31), or blood groups (p <0.21). However, educational level showed a highly significant association (p <0.0001) with *H. pylori* infection; 22 out of 32 (68.8%) illiterate patients tested positive as compared to 3 out of 9 (33.3%) university graduates. Interestingly, 30% of infected patients were receiving treatment and tested to follow up on the efficacy of the therapy while 70% of patients were untreated and tested for primary diagnosis. There were no statistically significant differences (p <0.47) between these two groups, with 14 out of 30 (47.6%) treated patients testing positive compared to 36 out of 70 (51.4%) untreated patients (Table 1).

**Figure 1:**
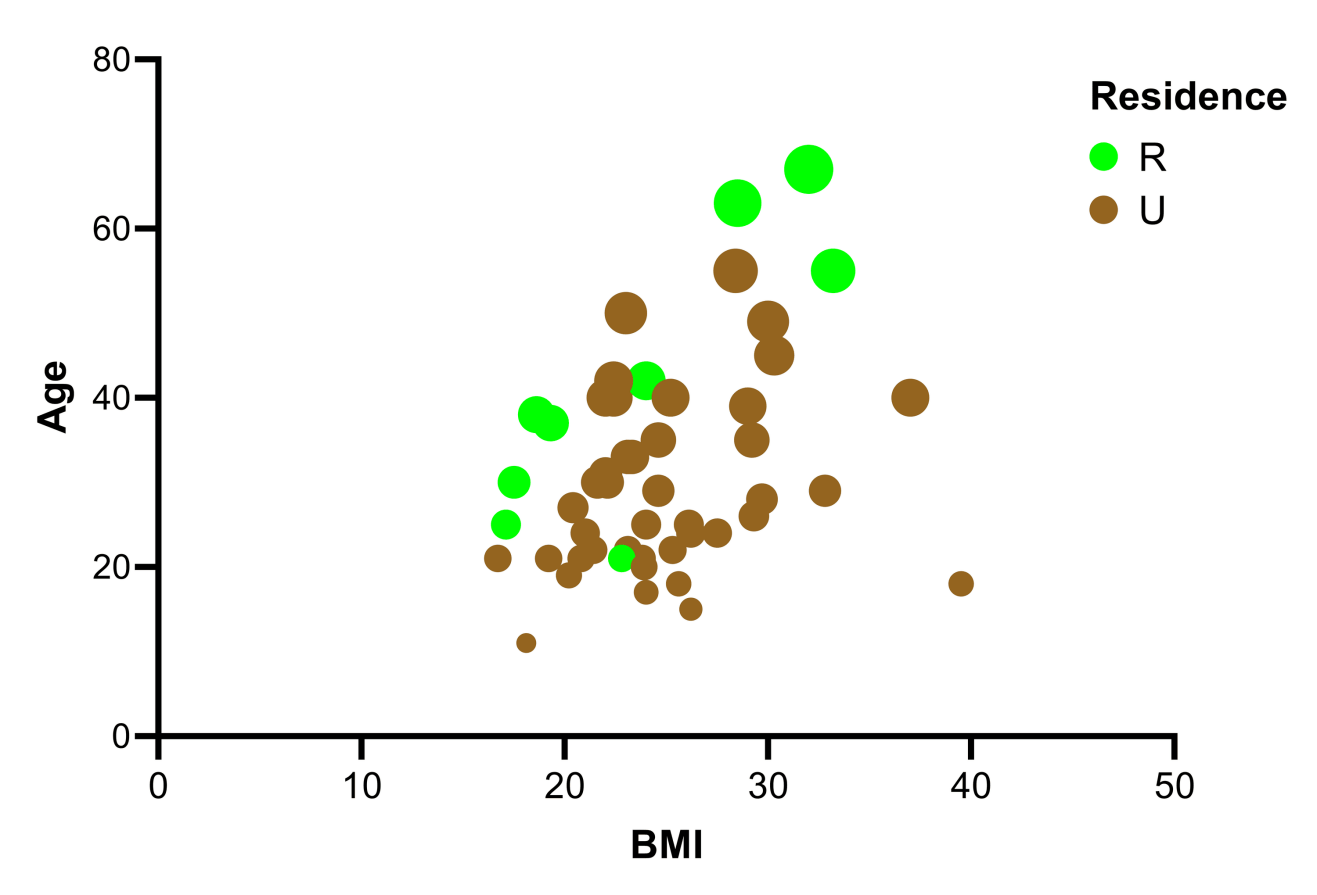
Correlation analysis between age and BMI of infected patients with Helicobacter pylori U=Urban, R=Rural, Brown bubbles represent “Urban residence”, light bubbles “Rural residence”; small bubbles “youngest infected”; and large bubbles “oldest age infected”

**Figure 2:**
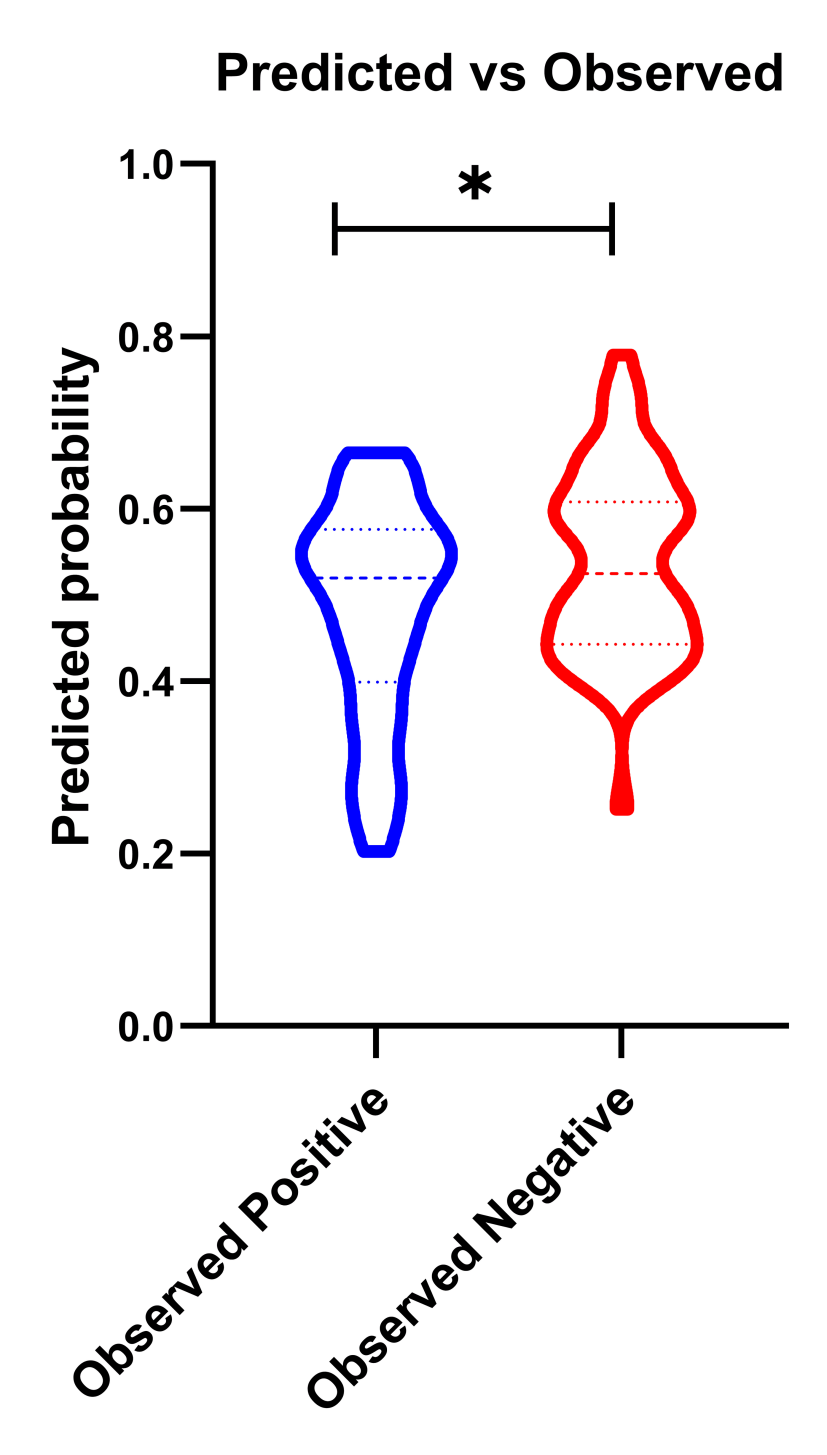
The logistic regression analysis of the predicted infections with Helicobacter pylori using the Hosmer-Lemeshow test

The initial statement suggests no significant association between BMI categories and *H. pylori* infection based on a chi-square analysis (p = 0.59). However, the scatter plot of the multiple linear regression analysis provides insights into the relationships between the BMI with both age and residence factors in these patients, highlighting a significant predictor of all potential importance for infections (P=0.0065) (Figure 1). This indicates that when BMI is considered in conjunction with other factors like age and residence, it shows a meaningful association with *H. pylori *infection. In addition, further analysis by multiple logistic regression suggests that age is only a significant factor (p = 0.0234) in predicting infections with younger (middle age) individuals being at greater risk than older patients using the Hosmer-Lemeshow hypothesis test (Figure 2).

## Discussion

The urea breath test has recently been confirmed as the gold standard non-invasive test for diagnosing *H. pylori*. It is highly sensitive, specific, and accurate as compared to other tests, for example, serology, stool antigen, rapid urease test, and culture [1]. The overall rate of *H. pylori *infection was 50.0% among symptomatic patients, as determined by the 14C-Urea breath test. This finding is consistent with recent laboratory-based studies in other cities in Iraq. For example, Sulaimani City showed a rate of 54.9% among dyspeptic patients [12], Erbil City showed 53.3% among gastroduodenal disorder patients [13], and internationally also Wuwei City, China, showed 53.0% [14]. In contrast, studies in Ramadi, Iraq, reported a rate of 68.7% [1], while in Duhok, the rate was 37.2% by culture and 68% by the rapid urease test [15], and among pediatric patients, the rate was 28% by the anti-*H. pylori* IgG test [16].

A significant variation in incidence rates was observed among different age groups in the study. There was a notable correlation between increasing age and higher infection rates. These findings are consistent with previous studies conducted in Iraq [17] and China [14]. However, they contradict a study conducted in Erbil, Iraq, which found no statistically significant association despite an increase in incidence with age [13].

There was no significant association between *H. pylori* infection and gender, similar to observations in Iraq [18], Turkey [19], and China [14]. However, a study in China reported higher infection rates in females than in males [20]. Discrepancies in these results could be attributed to factors such as study designs, sample size, methodology, participants’ lifestyles, socioeconomic status, sanitation, water supply, environmental conditions, and education level. No relationship was found between BMI and *H. pylori* infection in this study, consistent with findings from Jiangsu Province, China [20]. However, a study in Wuhan, China, reported a significant positive association between *H. pylori* infection and the risk of overweight/obesity [21]. While underweight patients had a higher rate of infection than those with normal BMI, the difference was not significant, likely due to their decreased nutritional status and immunity.

It is interesting to note that patients with *H. pylori* infection were significantly associated with low Hb levels. Recent studies in Northeast Romania [22] and Saudi Arabia [23] have also indicated that Hb and serum iron levels were significantly lower in *H. pylori*-positive cases compared to controls. The prevalence of iron deficiency was seven times higher in *H. pylori*-positive cases. Velayudhan et al. found that *H. pylori* has several iron acquisition systems that capture iron in the stomach lumen [24] while Kostaki et al. concluded that eradicating H. pylori improves the response to iron therapy in patients with iron deficiency anemia [25].

No significant correlation was found between *H. pylori* infections and other factors such as water source, smoking, family size, and blood group types. One possible explanation for this could be the sufficient chlorination of tap water. Similar findings were reported in China [20]. However, a study conducted in Northern Ireland indicated a link between smoking and *H. pylori* infection [26].

Regarding family size, this study disagrees with a study in Uganda, which reported higher infection rates with increased family size [27]. This difference is likely due to increased public awareness and hygiene practices during the COVID-19 pandemic. No association was found between *H. pylori* infection and different blood group types, similar to findings in Turkey [19]. However, this contrasts with a meta-analysis that found a significant association with blood group O in dyspeptic patients [28].

Higher levels of education were associated with better hygienic conditions. Approximately three-quarters of illiterate patients tested positive for *H. pylori* compared with university graduates. This finding aligns with the results from West Iran [29].

The current study found that 46.7% of symptomatic patients did not respond to triple therapy for *H. pylori*. The high failure rate of triple therapy can be attributed to several factors. One contributing factor is the emergence of resistant bacteria against levofloxacin, a drug commonly used to treat secondary bacterial infections in COVID-19 patients [30]. Furthermore, the widespread use of amoxicillin, often prescribed for various infections or even taken without a physician’s supervision, has accelerated the development of antibiotic resistance. Another factor may be the inconsistent treatment duration in our region, where some protocols adopt a two-week regimen, which may be insufficient to fully eradicate infections. In some cases of unresponsive patients, physicians sometimes recommend extending the course to three weeks.

Additionally, patients with high BMI also have higher failure rates [30]. It's worth noting that the three factors - age, residence, and BMI - have an important influence on the infected individuals with *H. pylori *in this study. While previous studies have focused on individual factors like age and BMI [14,17,20,21,30]. These variables are combined in a novel way in this study to give a more thorough knowledge of their combined impact. This comprehensive method provides a nuanced viewpoint that may help develop more potent *H. pylori *infection prevention and treatment plans.

This study has some limitations, including its focus on a specific geographic area and a relatively small sample size, which may not be representative of the broader population.

## Conclusions

This study highlights the significant prevalence of *H. pylori *infection among symptomatic patients in Zakho City as determined by the Helicoprobe 14C-Urea breath test. Notably, the prevalence is significantly associated with age and education levels. However, the triple therapy failed to eradicate *H. pylori* in half the infected patients. Among the studied risk factors, *H. pylori* infection was significantly associated with increasing age, low Hb levels, and illiteracy. Additionally, there was a significant association between *H. pylori *infection and combined increases in both BMI and age.
